# Relation between detection rate and inappropriate shocks in single versus dual chamber cardioverter-defibrillator – an analysis from the OPTION trial

**DOI:** 10.1038/srep21748

**Published:** 2016-02-19

**Authors:** Christof Kolb, Marcio Sturmer, Dominique Babuty, Peter Sick, Jean Marc Davy, Giulio Molon, Jörg Otto Schwab, Giuseppe Mantovani, Andrew Wickliffe, Carsten Lennerz, Verena Semmler, Pierre-Henri Siot, Sebastian Reif

**Affiliations:** 1 Deutsches Herzzentrum München, Klinik für Herz- und Kreislauferkrankungen, Abteilung für Elektrophysiologie, Faculty of Medicine, Technische Universität München, Munich, Germany; 2 Sacre-Coeur Hospital, Université de Montréal, Montréal, Québec, Canada; 3 University Hospital, Tours, France; 4 Hospital of the Order of St. John of God, Regensburg, Germany; 5 Département de Cardiologie et Maladies Vasculaires, Hôpital Arnaud de Villeneuve, Montpellier, France; 6 Cardiology Department, Ospedale Sacro Cuore, Negrar, Italy; 7 Department of Medicine, Cardiology, University Hospital, Bonn, Germany; 8 Ospedale Civile, Desio, Italy; 9 Piedmont Heart Institute, Atlanta, GA, United States of America; 10 Sorin CRM SAS, Clamart, France; 11 Klinik für Kardiologie und Internistische Intensivmedizin, Städtisches Klinikum München-Bogenhausen, München, Germany

## Abstract

The programming of implantable cardioverter-defibrillators (ICDs) influences inappropriate shock rates. The aim of the study is to analyse rates of patients with appropriate and inappropriate shocks according to detection zones in the OPTION trial. All patients received dual chamber (DC) ICDs randomly assigned to be programmed either to single chamber (SC) or to DC settings including PARAD+ algorithm. In a *post-hoc* analysis, rates of patients with inappropriate and appropriate shocks were calculated for shocks triggered at heart rates ≥170 bpm (ventricular tachycardia zone) and at rates ≥200 bpm (ventricular fibrillation zone). In the SC group, higher rates of patients with total and inappropriate shocks were delivered at heart rates ≥170 bpm than at rates ≥200 bpm (total shocks: 21.1% vs. 16.6%; p = 0.002; inappropriate shocks: 7.6% vs. 4.5%, p = 0.016; appropriate shocks: 15.2% vs. 13.5%; p = n.s.). No such differences were observed in the DC group (total shocks: 14.3% vs. 12.6%; p = n.s.; inappropriate shocks: 3.9% vs. 3.6%; p = n.s.; appropriate shocks: 12.2% vs. 10.4%; p = n.s.). The higher frequency of patients with total shocks with SC settings than with DC settings that benefit from PARAD+ was driven by a higher percentage of patients with inappropriate shocks in the VT zone (170–200 bpm) in the SC population.

The life-saving effects of implantable cardioverter-defibrillators (ICDs) are well established. However, the question on how best to reduce the frequency of inappropriate shocks associated with the therapy remains unanswered. Inappropriate shocks are thought to increase the risk of mortality and arrhythmias and are a cause of anxiety and reduced quality of life for patients^1^,^2^,^3^,^4^.

One strategy to reduce inappropriate shocks is to use dual-zone programming, providing antitachycardia pacing (ATP) as the primary therapy in the ventricular tachycardia (VT) zone between typically 170 and 200 beats per minute (bpm). Other strategies include delaying therapy until arrhythmias have persisted for a pre-defined number of cycles or seconds^5^,^6^. Dual-chamber (DC) ICDs which use atrial and ventricular intracardiac information to discriminate ventricular and supraventricular tachycardias (SVTs) should in theory be superior to single-chamber (SC) devices but this has been difficult to demonstrate in clinical trials. The jury remains out on the most appropriate ICD, algorithm and programming for ICD patients, including the most appropriate boundaries of the VT zone.

In the initially published OPTION trial (ClinicalTrials.gov-NCT00729703, date of registration: August 4, 2008)^7^ therapy with DC settings for ICD discrimination combined with algorithms for minimising ventricular pacing was associated with reduced risk for inappropriate shock compared SC settings, with no differences in morbidity or mortality between the two therapies. During a follow-up period of 27 months, the DC setting arm in OPTION showed superior results both on the time to the first inappropriate shock (p = 0.012, log-rank test for the differences between the groups) and on the percentage of patients who received inappropriate shocks (4.3% with DC settings v 10.3% with SC settings; p = 0.015). The percentage of patients who received ≥1 ICD shock was numerically but non-significantly smaller with DC therapy than with SC therapy (16.1% v 22.9%; p = 0.068). The rates of patients with only appropriate shocks were similar in both groups (11.7% v 12.6%; p = 0.790).

To provide a more differentiated picture of the patterns of inappropriate shocks in the two settings, we performed a *post-hoc* analysis of appropriate and inappropriate shocks delivered in the VT zone or the ventricular fibrillation (VF) zones in the SC and DC groups.

## Methods

The design and main results of the OPTION trial have been published^7^,^8^. In brief, this prospective, randomised (1:1), multicentre, single-blinded (patients), parallel-group trial enrolled 462 patients eligible for ICD therapy for primary or secondary prevention of sudden cardiac death (left ventricular ejection fraction ≤40% despite optimal tolerated heart-failure therapy).

After enrolment by physicians, random allocation sequence was requested by the investigator to the Sponsor. The 4-block permutation randomization list was generated by the study statistician using the proc plan procedure (SAS® software v9.2). Upon each request, the sponsor sent a closed envelope containing the assigned intervention for the patient considered. The envelope was opened by the investigator just before implant.

All patients received DC ICDs (OVATIO DR model 6550; Sorin Group, Milan, Italy) randomly assigned to be programmed either to SC settings (with the acceleration, stability, and long cycle search discrimination criteria activated) or to DC settings including the use of the PARAD+DC algorithm which differentiates supraventricular from ventricular arrhythmias in the zone between 170 bpm and 200 bpm. The SafeR™ mode (management of atrio-ventricular block) was activated in the DC group for minimised ventricular pacing.

In both groups, VT detection was programmed in the zone of 170–200 bpm. Any shock in this zone should be preceded by the delivery of 2 sequences of ATP. Ventricular fibrillation detection was activated at ≥200 bpm, with shock therapy preceded by 1 ATP for arrhythmias at heart rates between 200 and 240 bpm. Arrhythmias in the VT zone had to persist for 12 cycles and in the VF zone for 6 cycles before delivery of therapy in both groups. A slow VT zone was set at 120 bpm in both groups, to be used as a monitor zone for the SC setting group, whereas ATP with no shock was recommended in the DC group.

There were two primary end points: time to first occurrence of inappropriate ICD shock and the occurrence of all-cause death or cardiovascular hospitalisation. Rates of appropriate and inappropriate ICD shocks, all-cause and cardiovascular mortality, and cardiovascular hospitalisations were among the secondary end points. The average follow-up duration was 23.4 ± 7.9 months.

The current *post-hoc* analysis was carried out on the intention-to-treat population which consisted of 453 randomised patients, 230 in the DC group and 223 in the SC group. Rates of patients with appropriate and inappropriate shocks at two years of follow-up were obtained from the OPTION database. All reported shocks in the OPTION trial were validated by a blinded events committee of 5 experts who analysed the electrographic recordings in the device memories^7^. Data on shocks were obtained from the device records. For the analysis, the rates of patients with total, inappropriate and appropriate shocks were calculated for shocks triggered at heart rates ≥170 bpm and at rates ≥200 bpm, respectively in the SC and DC groups.

### Statistical analyses

Data were analysed using the SAS® statistical software package version 9.2. Continuous data are presented as mean ± SD. Proportions are presented as counts and percentages. Proportions were compared with χ^2^ test or with Fisher’s exact test when unpaired. When analysing the impact of programming the exact McNemar test was used for paired observations. Kaplan-Meier estimates of survival were calculated using standard methods. A two-sided probability <0.05 was considered significant.

### Ethics

The study protocol was approved by the ethics committee of the Technische Universität München, Munich, Germany (approval number 1549/06, 13^th^ June 2006) as the leading ethics board and additionally was approved by the local or national ethics boards of each participating centre. The study was performed in accordance with the Declaration of Helsinki, Good Clinical Practices and the applicable laws and regulations, methods were in accordance with approved guidelines and all study participants provided written informed consent prior to study inclusion.

## Results

The baseline characteristics of the OPTION study population have been reported previously (Table 1)^7^. The groups were balanced at baseline. The majority of patients were men and 75.3% had an indication for primary prevention of sudden cardiac death. Patients were well treated with pharmacological agents.

There were a total of 336 shocks during follow-up. Seventy-five shocks (22.3%) were recorded as inappropriate. Using a cut-off limit of ≥200 bpm, 245 shocks were recorded, 43 of which (17.6%) were inappropriate.

**Figure 1:**
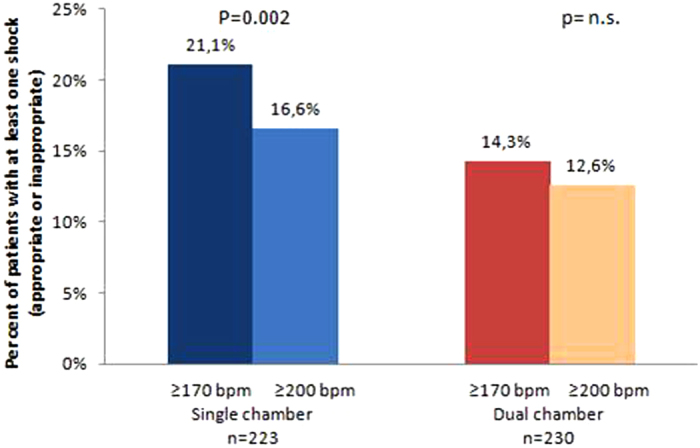
Percent of patients with at least one shock (appropriate and inappropriate) delivered at heart rates ≥170 bpm and ≥200 bpm respectively, in the SC and DC groups.

In the SC group the percentage of patients experiencing any shock delivered at heart rates ≥170 bpm (21.1%) was significantly greater that the percentage experiencing any shock delivered at heart rates ≥200 bpm (16.6%; Fig. 1; p = 0.002). Such differences were not observed in the DC group: 14.3% of patients experienced any shock triggered at heart rates ≥170 bpm compared with 12.6% experiencing any shock triggered at heart rates ≥200 bpm (p = n.s.).

**Figure 2:**
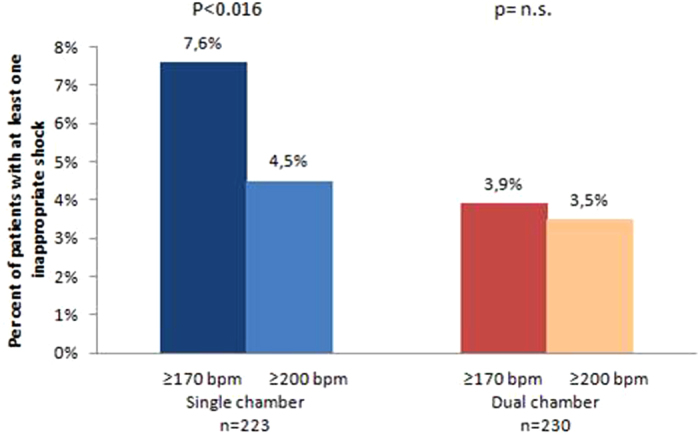
Percent of patients with at least one inappropriate shock delivered at heart rates ≥170 bpm and ≥200 bpm respectively, in the SC and DC groups.

In the SC group, 7.6% of patients experienced inappropriate shocks triggered at heart rates ≥170 bpm. However, 4.5% of patients experienced inappropriate shocks triggered at heart rates ≥200 bpm, a significantly lower frequency (p = 0.016; Fig. 2). In contrast, there were no significant differences in the percentages of patients experiencing inappropriate shocks in the DC group triggered at heart rates ≥170 bpm (3.9%) and ≥200 bpm (3.5%), respectively. SVTs are responsible for 93.8% of inappropriate shocks triggered at heart rates between 170 bpm and 200 bpm.

**Figure 3:**
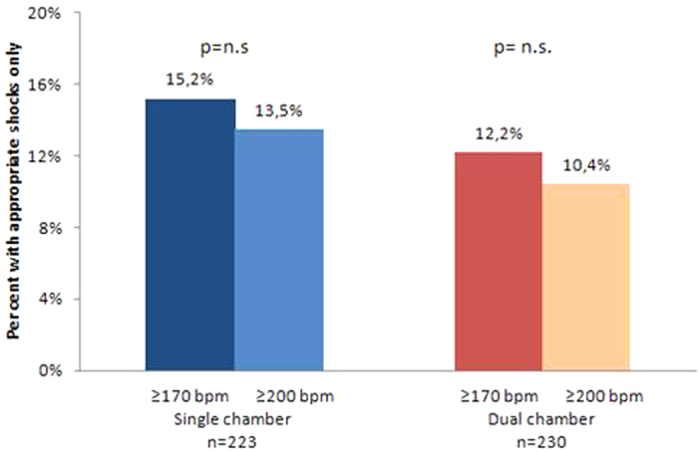
Percent of patients with appropriate shocks only delivered at heart rates ≥170 bpm and ≥200 bpm respectively, in the SC and DC groups.

The same analysis was performed for appropriate shocks (Fig. 3). In the SC group, 15.2% of patients experienced appropriate shocks triggered at heart rates ≥170 bpm compared with 13.5% using the ≥200 bpm cut-off limit. In the DC group, the percentages of patients with appropriate shocks were 12.2% v 10.4% triggered at heart rates ≥170 and ≥200 bpm, respectively.

**Figure 4:**
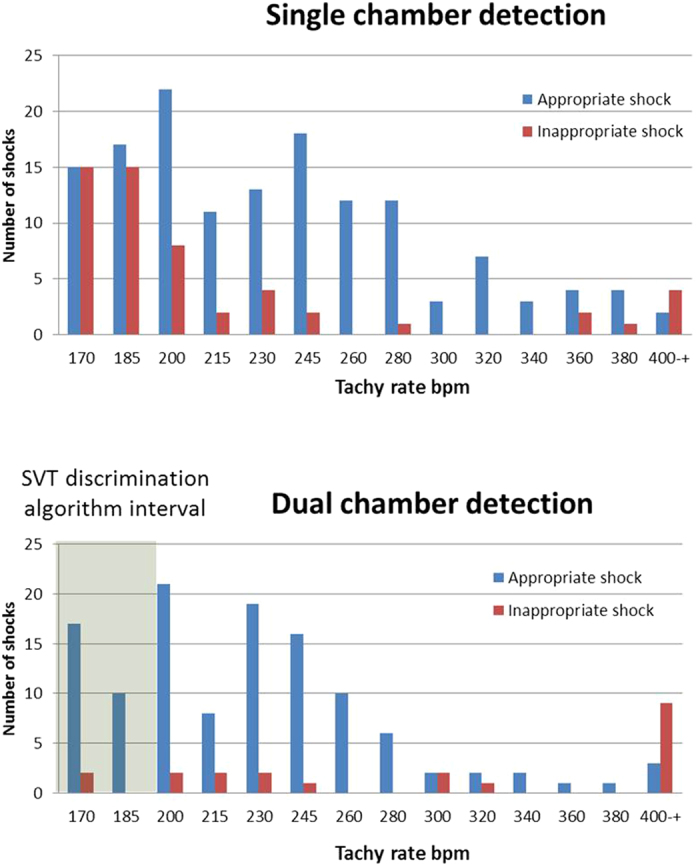
Rates of appropriate and inappropriate shocks in the DC and SC groups, respectively, triggered at different heart rates. The interval where the Parad+SVT discrimination was activated in the DC group is shaded.

A more granular analysis of shock rates triggered in different heart rate intervals supported the overall findings (Fig. 4). There were very few inappropriate shocks in the DC group at heart rates <200 bpm. At heart rates ≥200 bpm, there were few differences between the SC and DC groups.

**Figure 5:**
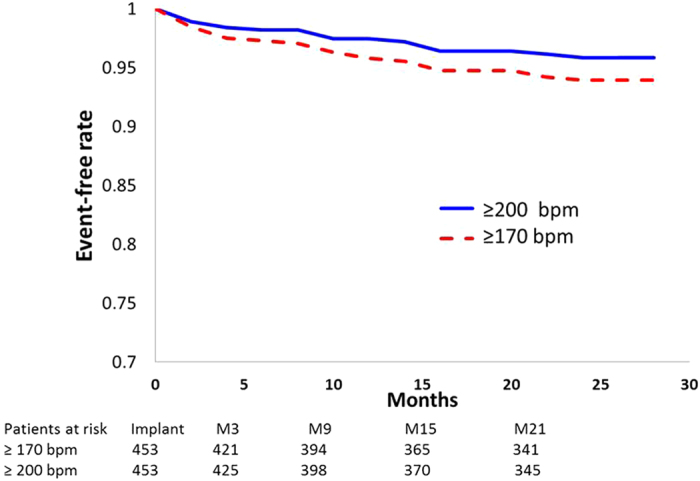
Kaplan-Meier analysis of rates of freedom from inappropriate shocks in the total population when measured using cut-off limits of ≥170 bpm and ≥200 bpm, respectively. Note that the two curves represent the same population of patients and not two different treatment arms.

The Kaplan-Meier curves of inappropriate shocks over time using the ≥170 bpm and ≥200 bpm cut-off do not suggest clustering of shocks at specific times throughout the two years of follow-up (Fig. 5).

## Discussion

In this *post-hoc* analysis of ICD therapies from the OPTION trial, we found significant differences in the potential effects of device programming on percentages of patients with inappropriate shocks between the SC and DC groups, respectively. In the SC group, when shocks triggered by events ≥200 bpm were counted, significantly fewer patients experienced inappropriate shocks compared with using a lower ≥170 bpm cut-off limit for triggers. In the DC group, the percentages of patients with inappropriate or appropriate shocks were not affected by changing the cut-off limits for triggering events. An analysis of shock numbers in different heart-rate intervals (Fig. 4) indicates that the ratio of inappropriate shocks in the SC group was higher in the intervals 170–200 bpm than at heart rates ≥200 bpm.

Our analysis indicates that a high cut-off limit may be beneficial for patients with SC devices, as has been suggested in the literature^5^,^6^. Programming higher cut-offs and longer therapy delays in the 170–200 bpm zone has been associated with lower rates of inappropriate shocks^6^,^9^. Conventional programming was further associated with very high rates of inappropriate antitachycardia pacing in the 170–200 bpm zone in the large-scale MADIT-RIT trial^6^. Such episodes are thus more likely to be erroneously classified as VT by the device algorithms. The OPTION study differs from MADIT-RIT in that it included a SC arm which could not be investigated in the MADIT-RIT study.

In addition to the higher cut-off limit, rates of inappropriate shocks were reduced in MADIT-RIT when devices were programmed with a 60-second delay at 170 to 199 bpm or when therapy was initiated at ≥200 bpm. The programming in OPTION for delays of 12 cycles in the VT zone and 6 cycles in the VF zone represented a short delay in comparison and it is conceivable that programming longer delays would have further reduced rates of inappropriate therapy.

Although the data from this *post-hoc* analysis should not be over-interpreted, we note that the Kaplan-Meier curves for rates of inappropriate shocks with the ≥170 and ≥200 bpm cut-off rates started to separate early and kept separating throughout follow-up. Based on the limited data available, the risk of inappropriate shock appeared to be constant over time, meaning that the increased risk of inappropriate shock with ≥170 bpm shock programming persists over time. This is comparable to what was observed in MADIT-RIT.

Whether it is advisable to disable shock therapy <200 bpm in SC devices cannot be decided on the basis of the current exploratory analysis. The risk of not receiving appropriate shocks when necessary remains to be accurately determined. In MADIT-RIT, no appropriate shocks were reported in the 170–200 bpm zone. However, an observation of low shock rates or no shocks in a randomised trial with less than three years’ follow up cannot be extrapolated to ICD recipients over longer time frames. Also, patients with documented permanent or persistent atrial fibrillation were excluded from MADIT-RIT, which probably reduced the risk of inappropriate therapies^6^. In OPTION, some appropriate shocks recorded were triggered at heart beats <200 bpm. The trade-off between lower risk of inappropriate shocks and potentially increased risk of not treating life-threatening events will need very careful consideration and the most appropriate cut-off point for SC ICDs remains to be established. For DC devices with shock-reduction algorithms in place, this problem does not arise. It may be also worth investigating whether raising the cut-off point further from 200 bmp would bring further benefits.

### Limitations

There are a number of limitations to the current analysis. First, it was a *post-hoc* analysis, with all the associated shortcomings, and from a modest-sized study population. Secondly, in line with other recent ICD trials such as MADIT-RIT, the number of patients with inappropriate shocks in the OPTION study was low which limits the power of the statistical sub-analysis. Thirdly, the programming choices including the delay periods reflect the historical nature of OPTION, which was initiated in 2006. However, both the lower ≥170 bpm and the upper ≥200 bpm therapy limits are in line with a number of other trials and recommendations^5^,^9^,^10^,^11^,^12^. Fourth, only Sorin’s ICD and PARAD+ algorithm were used and the conclusion may not be transferable to other devices and algorithms.

The OPTION population consisted of 75% of primary prevention patients and it is possible that the rates of inappropriate shocks differed between patients receiving an ICD for primary and secondary prevention. Given the modest size of the trial, a subgroup analysis of a *post-hoc* analysis was regarded as too unreliable to be of value and was not performed. Future prospective and adequately powered trials would need to be performed to provide data on this question.

## Conclusions

This *post-hoc* analysis from the OPTION study showed that the greater number of patients with shocks in SC arm, when compared to DC arm, was driven by a higher percentage of inappropriate shocks in the VT zone (170–200 bpm) in the SC population. There were no differences between the two therapies in the VF zone. The findings have potential implications for different programming strategies between DC and SC ICDs.

## Additional Information

**How to cite this article**: Kolb, C. *et al.* Relation between detection rate and inappropriate shocks in single versus dual chamber cardioverter-defibrillator – an analysis from the OPTION trial. *Sci. Rep.* **6**, 21748; doi: 10.1038/srep21748 (2016).

## Figures and Tables

**Table 1:** Baseline characteristics of the OPTION population.

Variable	DC group (n = 230)	SC group (n = 223)
Age [years], mean ± SD	62.6 ± 10.9	63.9 ± 10.0
Male sex, n (%)	186 (85.3)	189 (86.7)
Implant indication, n (%):		
• Primary implant prevention	168 (73.7)	171 (76.7)
• Secondary implant prevention	60 (26.3)	52 (23.3)
NYHA class I/II/III/IV, %	16%/62%/21%/1%	14%/67%/18%/1%
LVEF [%], mean ± SD	29.7 ± 8.5	28.3 ± 7.6
Cardiac disease, n (%):		
• Coronary	173 (75.5)	173 (77.6)
• Cardiomyopathy	79 (34.5)	84 (37.7)
QRS duration [ms], mean ± SD	111.0 ± 25.1	111.2 ± 28.3
Conduction disorders, n (%):		
• AV block	41 (17.9)	32 (14.3)
• Bundle-branch block	36 (15.7)	43 (19.3)
Atrial rhythm disorder, n (%):		
• Paroxysmal atrial flutter	11 (4.8)	2 (0.9)
• Atrial tachycardia	2 (0.9)	(2.7)
• Paroxysmal atrial fibrillation	24 (10.5)	25 (11.2)
Associated conditions, n (%):		
• Arterial hypertension	85 (37.1)	96 (43.0)
• Diabetes	48 (21.0)	53 (23.8)
Drugs, n (%):		
• Beta blockers	186 (84.9)	173 (82.0)
• ACE inhibitors/ARB	178 (81.3)	164 (77.7)
• Spironolactone	57 (26.0)	45 (21.3)
• Class III anti-arrhythmics	26 (11.9)	24 (11.4)

Differences between groups were not significant. ACE = angiotensin converting enzyme, ARB = angiotensin receptor blocker, AV = atrio-ventricular, LVEF = left ventricular ejection fraction, NYHA = New York Heart Association.
